# Health Literacy Studies Conducted in Australia: A Scoping Review

**DOI:** 10.3390/ijerph16071112

**Published:** 2019-03-28

**Authors:** Fahad Riaz Choudhry, Long Chiau Ming, Khadeeja Munawar, Syed Tabish R. Zaidi, Rahul P. Patel, Tahir Mehmood Khan, Shandell Elmer

**Affiliations:** 1 National Institute of Psychology, Quaid-i-Azam University, Islamabad 44000, Pakistan; fahad.riaz.psy@gmail.com; 2 Department of Psychology, Kulliyyah of Islamic Revealed Knowledge and Human Sciences, International Islamic University Malaysia, Kuala Lumpur 53100, Malaysia; 3 Pharmacy, School of Medicine, College of Health and Medicine, University of Tasmania, Hobart 7005, Australia; ming.long@bath.edu; 4 Faculty of Pharmacy, Quest International University Perak, Ipoh 30250, Perak, Malaysia; rahul.patel@utas.edu.au; 5 Department of Psychology, Jeffrey Cheah School of Medicine & Health Sciences, Monash University, Sunway City 47500, Selangor, Malaysia; 6 Department of Psychology, University of Wah, Punjab 47000, Pakistan; 7 School of Healthcare, University of Leeds, Leeds LS29JT, UK; s.t.r.zaidi@leeds.ac.uk; 8 Institute of Pharmaceutical Science, University of Veterinary & Animal Sciences, Lahore 54700, Pakistan; tahir.mehmood@monash.edu; 9 School of Pharmacy, Monash University Malaysia, Sunway City 45700, Selangor, Malaysia; 10 School of Medicine, College of Health and Medicine, University of Tasmania, Launceston 7250, Australia

**Keywords:** health literacy measurement, health knowledge, health numeracy, health-related literacy, consumer health information

## Abstract

Health literacy (HL) is an essential component of various literacies mentioned in the field of health and education, including cultural, technological, media and scientific literacies. It is important for motivating higher consumer engagement. We aimed to review previous studies of HL in Australia to inform future studies, extend current knowledge and further enhance HL. Using search strings, a systematic search of four databases (i.e., MEDLINE; Embase; CINAHL and Eric) was carried out. A Preferred Reporting Items for Systematic Reviews and Meta-Analyses (PRISMA) based search strategy led to identification of a total of *N* = 9696 records, that were further screened for inclusion in the review. The review findings were categorized into three major themes: (1) HL and health numeracy; (2) contrast of: knowledge deficiency, knowledge gained, problems of current health care system and (3) HL measurement methods and its domains. The findings from this scoping review show a dearth of measurement tools with sound psychometric properties for assessing HL. The findings also reveal low levels of HL in consumers which is in turn affecting health-related behaviors, utilization of health services and navigation of the health system. More recent developments have tried to integrate vital aspects, including introduction of applications to increase HL and exploring HL in Aboriginal communities.

## 1. Introduction

According to the World Health Organization, health literacy (HL) is the key determinant of the health and wellbeing of an individual [1,2]. It is one component of various literacies mentioned in the field of health and education, for instance; cultural literacy, technology literacy, media literacy and scientific literacy [3]. In the 1970s, the term “health literacy” emerged in the field of public health [4]. Since its emergence in 1970s, HL has been defined in multiple ways with varying conceptualizations [5]. HL is defined as the ability of a person to carry out health-related literacy [6]. Moreover, some studies define HL as an ability to perform various cognitive and social skills in order to gain, comprehend and utilize knowledge. HL is also considered as the ability of a person to improve health and well-being, and make decisions related with health care [5,7,8]. According to past research studies, HL has three levels: basic or functional level (skills to read and write in daily life); communicative or interactional level (social, cognitive as well as literacy skills); and critical level (able to use knowledge and deal with situations) [9,10]. The efforts continue to understand and define the mechanisms and effects of HL [11,12].

Much of the HL research originates from the scientific community in the United States and Canada, however, it has become more internationalized during the past decade [13]. HL is explored in many countries, such as Australia [14], Korea [15], Japan [16], the UK [17], the Netherlands [18], and Switzerland [19].

Even though extensive scientific literature exists on HL, most empirical studies use different terminology. There are some overlapping concepts defined by health and medical research, which has made it difficult to develop consensus and to take necessary steps in order to increase HL [9]. Likewise, efforts aimed at defining HL, have met with challenges because of the difficulty in measuring concepts such as interpersonal communication, quality, and an individual’s empowerment, motivation and ability to make decisions [10]. Similarly, there is a need to synthesize evidence on various constructs that have arisen from the domain of HL such as mental HL, sexual HL, and other context or disease specific health literacies as HL is likely to impact many facets of a person’s health [20,21].

Historically, research has predominantly focused on the functional HL of individuals [1]. This research established a relationship between HL and health outcomes and the ways that HL bestows individuals with skills for improving their health [11,22]. However, finding, comprehending and acting on health-related information does not depend solely on a person’s proficiency in these skills [11]. HL also requires the provision of easily understood, accessible and available information by healthcare information systems, as well as, the diffusion of understandable advice and information by the health service providers [23].

The Australian government, together with other countries, has made it a priority to address HL as it is recognized as a vital aspect of better health and higher quality of care [24,25,26,27]. Australia has taken a national approach for HL [28] since HL was first prioritized and included in Australia’s national health goals and targets in 1993 [29]. The Australian Government has carried out various activities within and outside the health system in order to address HL (for instance activities aimed at health promotion, health protection, disease prevention, health care maintenance, and systems navigation), but maintenance of such efforts and measuring their success has proven to be challenging [30]. It is difficult to design a general set of targets that reflect the social, economic and environmental elements of health in conjunction with more customary measures of health status. Furthermore, there is a need for inter-sectorial action to address the determinants of health [30]. Subsequently, Australia launched a program of health reforms in 2010 for betterment of effectiveness, competence, suitability and availability of health-related care [25,31,32,33,34,35]. In line with this, the Department of Health and Human Services in Tasmania formulated a plan to highlight the government initiatives for increasing HL [36]. Around the same time, a hospital located in South Australia launched a program to recognize those aspects of hospital which facilitate or hamper abilities of patients in utilizing hospital facilities [37].

According to the Australian Bureau of Statistics [30] the prevalence of low HL in Australian adults was 60% which raises concerns about the capacity of people to effectively participate in their own health care and engage with health services [30]. A total of 59% of 17 to 74 years old individuals did not have adequate HL skills to effectively and efficiently understand and apply health-related information in their daily lives [30]. Research revealed that only 56% of the Australian population had insufficient general literacy in order to deal with daily life activities and work [36]. Almost half of the adult populations in developed countries such as the US, New Zealand and Canada have inadequate HL skills [38,39,40,41,42].

Even though there is increasing prevalence of chronic diseases, the levels of HL continue to be low in the United States and Canada [11,43,44]. Indeed, there is a gap in our current understanding of HL and its relationship to chronic diseases [10,45] but it is definite that people with low HL have worsened health outcomes [46]. The overall estimates revealed that there are 1.5 to 3 times greater chances of worsened health outcomes among individuals with low HL [47]. The low levels of HL are linked with greater utilization of services of health, less information among consumers, and worsened health outcomes [22]. The underlying access and equity issues can be exacerbated, for disadvantaged or vulnerable groups in Australia, due to low HL [48]. The Australian Bureau of Statistics revealed that according to Ethnic Communities’ Council of Victoria, compared to 43% of individuals born in Australia, only 33% of the individuals born outside Australia have sufficient or higher HL [49].

The HL of a patient has a strong influence on the extent of a patients’ medication adherence [50,51,52,53,54] and associated health care utilization [55,56]. HL is a known predictor of medication adherence [50,57,58]. Patients with low HL had been shown to experience a higher rate of medication errors and incorrect interpretation of medication label warnings [59,60,61]. The patients with low HL levels are difficult to counsel because they cannot comprehend the benefits and risks of medications adherence on health outcomes [62,63].

In Australia, the provision of safe and quality healthcare have been impacted by poor sources of health information, low HL across the population, and rising demands from a complex health system [30,64]. For example, a research study in Australia showed diminished cognition, depression and insufficient HL as the main risk factors for patients on warfarin therapy [65]. The rising costs linked with low HL have been examined in a systematic review and extra costs have been seen to correspond to almost 3–5% of aggregate health care expenditure [66].

The process for reviewing, and collecting and synthesizing evidence from previous health information research varies [67]. For instance, scoping reviews are gaining popularity to review health information research [68,69]. The reviews vary on three main dimensions. The first dimension is the aim of a review, its theoretical and ideological premises, and its logics of aggregation and synthesis of data. The second dimension consists of structure, quantity, as well as mapping and synthesis of the research and the data connections between the data. The third and an important dimension is the profundity and the extent of ‘work done’ in exploring a research issue [70]. A scoping review was chosen for this task of reviewing the research about health literacy in Australia. A scoping review enables the initial assessment of potential size and scope of available research literature. It also helps in recognizing the nature and extensiveness of research evidence [67,68,70]. In this present study, the scoping review helped to identify the boundaries of HL within the context HL related work undertaken in Australia. Contrary to narrative or literature reviews, scoping reviews require analytic re-interpretation of the literature [69]. However, a potential limitation of the scoping review is the risk of bias because unlike a systematic review, usually a quality assessment is not conducted in scoping reviews [71,72,73]. This lack of quality assessment may limit the uptake of findings to policy and practice [67] as the present scoping review included a vast range of published as well as gray literature on both HL and its various sub-fields.

This scoping review aimed to explore the prevailing awareness regarding HL in the published literature in Australia. Furthermore, we aimed to understand the prevailing definitions and concepts of HL and constructs, a lack of standardization in measurement tools and agreement on what needs to be measured (e.g., key outcomes), as reported in the Australian studies, for developing an understanding around assessment of HL, its domains and constructs.

### Significance of the Study

This scoping review augments current knowledge on HL, offers further clarity in various concepts related to HL, and highlights how HL has advanced both temporally and spatially in Australia. This review attempts to synthesize studies directly focusing on HL, as well as various constructs that have arisen from this domain. An analysis of these constructs under the umbrella term of HL will enhance clarity on how HL fosters agency for healthcare decisions and navigating the healthcare system within the context of a person’s life. This scoping review to analyze the research on HL is necessary for the following reasons. First, variability exists in the scientific community about the conceptual understanding of HL and its terminology, leading to different interpretations of HL in health and medical research. Second, there are some difficulties in assessing concepts related to HL such as, interpersonal communication, quality, individual’s motivation, empowerment, and decision making and its constructs. Third, low HL or lack of adequate levels of HL have been linked with various negative outcomes, such as, greater utilization of services of health, low medication adherence, less information in consumers, equity issues for disadvantaged groups of Australia, and worsened health outcomes. In response to these aforementioned reasons, this review aims to review, analyze, and summarize previous research studies undertaken in Australia so that future studies can build on the current knowledge to further enhance HL.

## 2. Materials and Methods

Arksey’s and O’Malley’s [74,75] approach for scoping reviews was used. This process for review helps the researchers in examination of the nature and range of research carried out on a specific topic, helps in finding gaps in previously available literature on a specific topic, and analyze the value of carrying out extensive systematic reviews [74,75]. The five stages of scoping review performed are explained below.

### 2.1. Identification of the Scoping Review Question (Inclusion Criteria) (Stage 1)

The research question was identified by noting the HL variables and relationships between HL and multiple factors associated with it in previous studies. Thus, the scoping review question was framed as: What do the previous and current literature explain about the relationship(s) between HL and: (1) concepts and key outcomes of HL; (2) standardization and assessment of HL; and (3) awareness of HL.

### 2.2. Identification of Relevant Studies (Stage 2)

To identify studies fulfilling the inclusion criteria, four databases were searched: MEDLINE; Embase; CINAHL and Eric (Table 1). A systematic literature search strategy was adopted to determine the appropriate studies regardless of theoretical foundations they were affiliated with. For every database, search terms to identify studies included the following: HL (such as, numeracy, literacy, consumer health information, medical literacy, health education, health knowledge, educational status, and names of health literacy instruments), treatment decision (such as, information, awareness of risk, perceived barriers and benefits), mental HL (Mental health, mental illness*, mental disorder*, mental disease*, depression AND literacy/*health literacy), sexual HL (sexual health or sexual health or reproductive health or sexual and reproductive health or maternal health or maternal welfare or neonatal health or perinatal care or perinatal health or prenatal care/ or prenatal health) and Australia*. These key terms were extracted in order to capture the three levels (i.e. basic or functional level, communicative or interactional level, and critical level) of HL and its constructs [9,10]. We combined the search terms with Boolean operators “OR” and “AND”.

**Figure 1:**
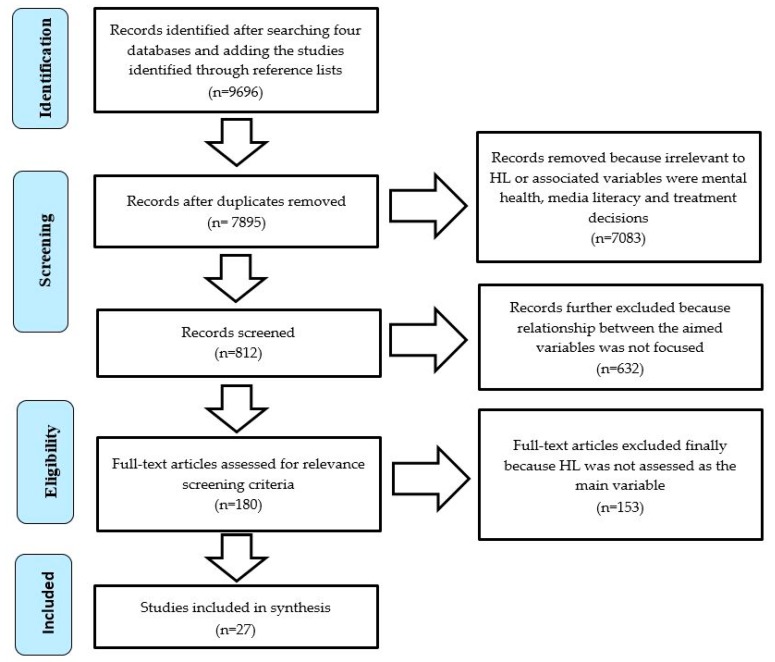
PRISMA flow chart for search process.

These databases retrieved 9696 records (as shown in Figure 1). The reference lists of all the review papers were also searched manually and an additional 12 records were found. The articles that directly mentioned the terms relating to HL, and treatment decision in title or abstract were prioritized. After excluding the studies based on our inclusion criteria and removing duplicates 27 records remained.

### 2.3. Study Selection (Stage 3)

All the authors of this scoping review unanimously formulated the screening criteria comprised of two levels. These criteria were later on applied and initially tested on a small number of records. The first screening criterion was to assess the title as well as abstracts of every record by authors. KM and FRC read in detail all the records that remained after first screening criteria for relevance.

The second criterion helped in deciding whether or not to exclude those studies which did not directly mention HL in the title, abstracts and results section. The studies only mentioning HL in the discussion section were excluded. Figure 1 (Preferred Reporting Items for Systematic Reviews and Meta-Analyses; PRISMA) shows detail of screening procedure. Three authors mutually settled all conflicts that emerged during this process of screening. The inclusion and exclusion criteria developed for this review are shown in Table 2. The PRISMA checklist is available as Supplementary File.

### 2.4. Charting the Data (Stage 4)

A data extraction chart was developed by KM which was later on assessed independently by co-authors FRC, LCM and TMK on five records. After this initial assessment, KM applied this data extraction chart on the rest of the studies. The data extraction chart consisted of information related to the first author and year, study objective, study population and study design.

### 2.5. Collating, Summarizing, and Reporting the Results (Stage 5)

The objective, study population and study design are described in the Table 3. The temporal and geographical distribution narrative account of all the included studies is included in the Table 4.

The empirical relationships relevant for answering the research question are reported and thematically organized. The first author analysed, organized, and synthesized the data comprehensively through thematic analysis [105]. The analysis was then discussed with all the co-authors and the categories were identified. Approximately, half of the thematic analysis was autonomously carried out by the two authors and all differences were settled through discussion between two and all the authors. The results are presented descriptively.

It is not an integral component of scoping review to appraise the quality of included studies, [74,75]. The limitations in method section of every record, gaps present in previous literature, their implications for further research, formulations of policies and in practice were highlighted in this review.

## 3. Results

A total of 9696 records were identified after searching four databases and adding the studies identified through reference lists. After removing duplicates, 7895 records were left. Out of which 812 records were selected for relevance screening criteria and eligibility. The articles that did not measure or focus on HL as the main variable and its relationship with other variables were excluded. Finally, 27 records were retained for this review.

### 3.1. Research Designs and Study Populations

The qualitative studies were the most prevalent (*n* = 7), followed by experimental (*n* = 6), and cross-sectional studies (*n* = 7). There were two (*n* = 2) letters to editor and two (*n* = 2) mixed-method studies. There was one each of the following research designs: prospective quasi-longitudinal study, omnibus survey, and telephone survey. Across these studies, the number and socio-demographic characteristics of the participants recruited by the researchers varied. For instance, women aged at least 18 years who were admitted to the postnatal maternity ward at a hospital, people with schizophrenia, general practice attendees; young Australians aged 12–25 years, and South Australian rural and urban young men aged between 15 and 30 years.

Out of all the 27 studies, five studies were carried out in different areas of Victoria [86,88,92,98,99], five in New South Wales [83,89,91,101,103], five in Queensland [76,93,95,100,104], three studies in Western Australia [81,85,90], two studies in South Australia and Australia Capital Territory respectively [77,78,80,94], and one study each in Tasmania and the Northern Territory, and one study recruited participants from all of the states and territories except Tasmania [70,72,73]. One study recruited participants from across Australia [79], and one study was carried out among Indigenous Australians [87]. The present review also brought forth the time sequence of all the included studies related with health literacy. Three studies were published before the years 2010 [77,79,87] and 25 studies were published between 2010 and 2018 [76,78,80,81,83,85,86,88,89,90,91,92,93,94,95,96,97,98,99,100,101,102,103,104]. The spatial and temporal sequence demonstrates that health literacy research is Australia is widespread and most studies have been published since 2010. The majority of the health literacy research published has originated from studies in Queensland, New South Wales and Victoria.

We identified three main themes from the collected data: (1) HL and health numeracy (2) HL measurement methods and its domains and (3) HL as knowledge deficiency, knowledge gained knowledge of current health care system. Knowledge deficiency, knowledge gained, problem of current health care system were the focus of 20 studies, and 9 studies contrasted HL with health numeracy. HL measurement methods and its domains were common to all of the 27 selected articles and this theme relates to our review question about the standardization and assessment of HL. The spatial (geographical location) and temporal (time sequence) of HL research conducted was analyzed in all the included papers. The thematic analysis will be discussed in more detail below.

### 3.2. Theme 1—Health Literacy and Health Numeracy

Research studies included in this scoping review explored the associations between HL and health numeracy and health outcomes. The studies defined and interpreted HL differently as well as sexual-, mental and oral- HL [75,76,77,80,81,86,87,88,91,96]. One study used an adapted framework called Paasche-Orlow and Wolf’s model of the pathways linking health literacy and health outcomes [97]. Furthermore, a study showed that despite having knowledge about pregnancy and weight gain, some women experienced excessive gestational weight gain, indicating a need to provide information regarding nutritional ranges and dietary intake portions [76]. The effect of various diseases on health numeracy and literacy of children, as well as the caregivers, was highlighted by the research studies [69,89].

The research studies reviewed revealed that hospitalization, medication adherence and health numeracy were strongly linked. One study proposed that it is vital to assess health numeracy as it can increase the risk of preventable hospital admissions, for example hospitalization due to asthma [77]. Another research study found that medication adherence in patients with osteoporosis was dependent patients’ understanding and appraisal of the risks and benefits of a treatment [88]. Similarly, a research study showed that patients made informed decisions if they were acquainted about risks and benefits of various bowel cancer screening procedures [91]. According to the findings, adequate health numeracy assisted patients in calculating cumulative outcomes of the procedures, estimation of efficacy and calculation of percentages of deaths due to absence of such procedures [91]. In the same way, in order to increase HL and health numeracy of their patients, radiation oncologists used a number of approaches to communicate complicated information regarding appointments and medicines intake [83].

### 3.3. Theme 2—Health Literacy as Knowledge Gain, Knowledge Deficiency and Knowledge of Current Health System

This theme emerged from the numerous ways that researchers endeavored to ascertain gains in health literacy knowledge about particular health conditions and the health system. For example, in a study aimed at quantifying functional HL in asthmatic adults and assessing relationships with health status and morbidity [77], findings revealed that incidences of at-risk HL was approximately 25% and of inadequate HL was around 21% [77]. Participants with chronic asthma reported 15% inadequate HL [77]. Another study highlighted the knowledge deficiency in the primary healthcare system of Australia and misperception regarding differences of the roles of nurses. The findings revealed the inadequacy of health-related knowledge (HL) regarding how to utilise the health system [96].

Studies explored the social and cultural factors that impact on health-related knowledge. When the HL of chronic low-back pain patients and non-chronic low-back pain patients were compared, these main findings emerged: *chronic low-back pain patients had lower scores on domains measuring attitudes towards health, family errands were the reasons for not performing health-related behaviors, absence of effective coping impeded engagement in health-related behaviors, and exacerbated pain and emotional suffering* [90]. A study about knowledge, perceptions and experiences of chronic hepatitis B revealed that Aboriginal respondents had a deficiency of health-related knowledge and exhibited the wish to increase hepatitis B health literacy [73]. Participants in this study also identified the need for translated information that incorporated the cultural context in order to effectively transfer information [73]. In addition to communicating in culturally suitable languages for patients from diverse backgrounds, visual aids were acknowledged as an important means for enhancing transfer of information [97]. Another study revealed how the absence of motivation on part of consumers played a role in low health-related knowledge as consumers that were aware of their deficiency of health-related knowledge, expressed little motivation or eagerness to improve it [96].

These studies also examined the way that patients find and use health-related information. For example, patients with chronic low back pain were challenged by finding and utilizing health-related information. The findings suggested that the HL of chronic low back pain patients seemed to be directly influenced by how they managed themselves, although these patients easily understood information related to health [90]. Aboriginal study participants with chronic hepatitis B and their community members misunderstood information regarding the cause and transfer of hepatitis B [73]. These misconceptions resulted in stigmatizing attitudes among patients and community members [97].

The studies included in this scoping review identified the ways that the health literacy skills and experience of service providers influenced the health literacy of their patients. For example, the language (complexity and dialect) used was reported as a challenge and a significant barrier towards understanding health-related information and knowledge [97]. The findings also revealed that healthcare providers may lack knowledge and have an inadequate understanding of health-related information [73]. Gender was also highlighted as a factor which influenced the transfer of information whereby this was more effective if healthcare providers and patients belong to the same gender as the healthcare provider [97]. Another research study revealed that historical legacy greatly influenced oral health practices, access to oral health services and health-related decision making of individuals [87]. The health literacy of the future health workforce has also been studied. For example, students who were enrolled in a medical or nursing disciplines had highest levels of sexual HL [70]. The findings from this study also showed that when scores of Australian/New Zealander students were compared with overseas-born students, all the overseas-born students had lower scores on sexual HL [102].

The research studies included in this scoping review evaluated the impact of various interventions designed to increase health literacy such as the role of workshops; non-medical providers of health care; activities of schools; awareness campaigns to increase HL levels in consumers; the need to change structure and policies in schools to achieve health outcomes; and the influence of functional, interactive and critical health literacy on health-related decision making [89,90,94].

Within the research studies included in this scoping review, there was a particular focus on mental health which showed a rise in levels of mental HL in rural and urban community. This was evident in the following findings: better understanding and prevention of mental health issues; approval of seeking help from mental health care providers; preferring to access online sources of information; and consulting doctor, or talking to peers or family members about a mental health issue [78,79,80,85,97,101,104]. However, lack of awareness regarding mental health was profound in Aboriginal men who lived alone which was related to not discussing problem within the community, and use of technical language by the health care providers [78]. These studies also explored social and cultural factors that influence mental HL such as the influence of parental beliefs on the choice of resources young individuals use to gain mental health information [79]. Cultural influences were evident in studies that explored perspectives of the western medical model regarding etiology and treatment of mental problems; and the need to educate refugee communities from Iraq and Sudan about etiology and treatment of mental disorders [73,76]. Another research study highlighted the way that health awareness programs were not beneficial for the members of an Aboriginal community because the health messages were targeted at mainstream Australians [78].

The research studies included within this theme of HL knowledge investigated HL within healthcare systems. These investigations included the ways health care professionals assessed and responded to the HL of their patients. For example, a research study conducted with radiation oncologists showed recognition and acceptance of varying levels of HL in their patients. This study used different subjective approaches to analyze HL levels in the patients, however, those oncologists were against the notion of incorporating literacy screening in practice [83].

Another study revealed that caregiver’s HL about cancer was multidimensional concept which was based on individual and social aspects; and affected by multiple factors within the community including the healthcare system. This study argued for system-level change through exploring factors that influence HL of caregivers in order to help the healthcare providers, as well as, policymakers to adequately design information, communication and education approaches to meet caregiver requirements [98].

The Australian government has implemented policies and programs to enhance health-related knowledge, particularly through the design of Aboriginal health service delivery models, however, these initiatives have had mixed success. A study on remote dwelling Aboriginal Australians, highlighted the use of technologies especially mobile applications to improve the HL across multiple settings [97]. However, clinical systems for conducting health checks were unclear to staff, and there was a lack of clarity about staff responsibilities for initiating and conducting the health check. Additionally some staff perceived the some of the health check content as sensitive, invasive, culturally inappropriate and of questionable value [93]. A study on older patients taking warfarin revealed limited health literacy [103].

### 3.4. Theme 3—Health Literacy Measurement Methods and Its Domains

The current review included research studies which mentioned the names of tools used to assess HL, its domains or constructs. The included studies also contained other instruments administered for various purposes, but our aim is to focus on those tools which are used to assess HL, its domains or constructs. For instance, one research study used the “*Newest Vital Sign*” to assess the functional domain of HL in South Australian asthma adults [77]. In the same way, another study mentioned “*Health Literacy Measurement Scale (HeLMS)*” in order to assess wider aspects of HL. According to the study, the scale has various domains targeting patients’ health attitudes, understanding and using information related to health, financial conditions and support system, healthcare services provided by general practitioner, interaction with healthcare provider, and being farsighted regarding management of health issues [90]. Similarly, a timed test, targeting reading comprehension, named as “*Shor*t* test of Functional Health Literacy in Adults (S-TOFHLA)*” was administered in another study [95]. The “*Health Literacy Questionnaire (HLQ)*”, developed from the HeLMs, was also favored as a HL measurement tool that accounts for a broad range of social and relational factors which impact an individual’s HL needs [88,103].

Other studies explored the associations between health literacy and health seeking behaviors. For example, one study explored the associations between health conditions, utilization of health services and academic performance using the “*School Entrant Health Questionnaire*” [99]. Two instruments were used in a research study which targeted sexual HL; the “*ARCSHS Secondary Students and Sexual Health Survey*” having two domains (knowledge and HIV/Hepatitis), and “*University of Missouri Sexual Health Survey (SHS)*” having three domains (knowledge, sexually transmissible infection and pregnancy) [102]. A study aimed at assessing the efficacy of workshop in increasing levels of mental HL utilized two separate instruments; “*First Aid Knowledge Test*” for evaluating literacy of participants regarding various strategies of aid, and “*Mental Health Literacy Questionnaire for Bulimic Type Eating Disorders*” to assess HL related to eating disorders [94]. However, the psychometric properties of the HL tools with respect to the population investigated in each study or the HL tools in general were absent.

Various qualitative studies used semi-structured interviews and focus group discussions to explore the broader aspects of HL such as: patients’ literacy regarding nurses’ role; patients’ HL and the need to acquire information regarding hepatitis B; mental HL and help seeking behaviors of individuals belonging to an Aboriginal Victorian community; changes in oral HL, as well as causes and prevention of poor oral health; barriers and facilitators that influenced health seeking behaviors; literacy regarding mental health issues; HL of radiation oncologists regarding patients’ comprehension levels; and strategies utilized by radiation oncologists to raise HL of patients [83,86,87,93,96,97,101].

Some research studies directly measured HL through randomized control trials or using tools but did not mention the names of those tools. For instance, one study assessed mental HL through a questionnaire based on symptoms of depression, as well as through an experimental design which involved giving knowledge regarding nature and appraisal of intrusive thoughts. In another study, trust and change in levels of HL due to various audiovisual and electronic means of gaining information was assessed through a questionnaire; and in another study, a self-reported survey was used to collect data regarding HL and various health related aspects of new mothers [76,78,80,85].

Questionnaires and survey tools were used to assess aspects of health literacy relating to knowledge of health and health systems. A questionnaire-based assessment of informed choice and knowledge was carried out to explore the role of decision aids in HL; instrument-based assessment of clinical trial literacy related with hepatitis C virus; and the Delphi method to assess health information system [106]. Other questionnaire tools were used to assess the competencies of low and middle income countries; develop and assess a model of HL for caregivers of cancer; mental health literacy was assessed with a computer-assisted telephone survey as well as interviews; and a randomized controlled trial was used for assessing the usefulness of a first aid linked to mental HL [79,81,89,91,98,100].

## 4. Discussion

The large number of empirical studies found in the literature to date shows the importance of HL in medicine and public health. Additionally, interest in the issue is on the rise in Australia and other parts of the world. Past empirical studies on HL in Australia point to the need to enhance the quality of health communication and to integrate HL into health professional care practice and service delivery. This review provided significant insights regarding the status of HL in Australia, as well as the areas which need further investigation from the research community.

This review sought to examine prevailing definitions and conceptualizations of HL, identify standardization of measurement tools of HL and key outcomes, and current awareness regarding HL in Australia. The review also highlights the geographical and temporal distribution of HL studies conducted in Australia and shows that limited research studies have been published among Indigenous Australians, and in some states and territories. The review has shown that research is needed to construct and validate instruments for measuring HL and its key components. This study has also highlighted the presence of low HL in consumers of health care services, misconceptions regarding various aspects of diseases, treatment and healthcare system, and need for longitudinal, mixed methods and qualitative research designs exploring complex causal mechanisms that influence relationship between HL and health outcomes. To attain maximum health gains, there is a need to increase HL of consumers and implement strategies for HL screening as a component of the clinical practice of health professionals.

There are two contrasting approaches to describe HL, either as a polarized or a complex phenomenon with its own features that specify HL or the absence of HLin variety of forms and situations [107]. According to the polarized approach, HL is a permanent state which cannot be improved. However, the complex approach views HL as a dynamic phenomenon, whereby HL varies and depends on the state of the person, the situation, the culture or the environment of that person. The findings of this review also highlight that the complex approach is consistent with research on health outcomes that replaces e formerly accepted and understood knowledge [107]. A dynamic and complex view of HL may be more receptive to broader perspectives that may impact health of a person. The tools for measuring HL and key outcomes are discussed in the review [77,88,90,102] and show the need to develop standardized means of assessing this construct.

Furthermore, complex causal mechanisms influence the relationship between HL and health outcomes. Such causal mechanisms are shown to be affected by different contextual, individual and external factors for instance age, educational levels, financial status, cultural background, social support, and the media [108,109]. Likewise, various models have been proposed to highlight these links [5]. Such models have limited efficacy as there is a dearth of supporting data, moreover, such models have been found to underestimate existing complexity of the elements, associations and interactions [25,35].

A number of factors play roles in weakening the findings of prevailing research studies, such as: (1) Very few studies examined the underpinnings of complex causal mechanisms which influence the relationship between HL and health outcomes [110,111]; (2) Absence of instruments targeting HL and its domains; (3) Significant dearth of studies evaluating current strategies and technologies aimed to improve HL; and (4) A lack of research studies directly analyzing health numeracy. The knowledge deficiency highlighted in HL studies [77,90,96], problems of present health care system [97,98,100], and inadequate levels of health numeracy [76,80,88,91,104] must be taken into consideration and efforts should be made to increase HL knowledge of the consumers as shown by the studies [78,79,80,85,97,101].

The World Health Organization reported a decline in human and economic resources in health systems due to low levels of HL [27]. Prior research has shown HL to be an independent predictor of information of a patient regarding his/her chronic illness and such low levels of HL prove burdensome for the health care industry as patients are not well equipped for self-care including medication management [10,112]. Low HL was found to be associated with declines in level of health knowledge, poor health outcomes, increased vulnerability, unhealthy behaviors, lower utilization of preventive services, worsened status of health, decline in usage of mammography, decline in uptake of vaccination for influenza, rise in rates of hospitalization, poor adherence to medication regimen, increased chances of death at old age and poor health status in old people [22,46,47,84,112,113,114,115]. Likewise, poor HL in adults has been shown to link to low levels of understanding of information about health and disease, difficulty with self-care management, and increased rates of chronic disease morbidity and mortality [22].

Functional HL skills are shown to be unrelated with the confidence and skills of a person to engage in a conversation with health professional, rather the person’s perception of their social position, has been linked with consultation with a health professional [116]. The qualitative studies carried out among patients and health professionals reported a number of important abilities to find, comprehend and utilize health-related information [117,118]. These comprise of awareness regarding when and where to gain health-related information, ability to recollect and process the information, and necessary skills in order to act upon this information. A research study proposed that HL progresses with time and is a staged process of gathering health information, fostering skills and practices related with health, carrying out actions and making informed decisions on the basis of options available [119]. The results show improved information and higher involvement in decision-making [119].

Health care is continuously changing and the responsibility for its rising demands are placed on both individuals and professionals to comprehend and keep abreast with research in the health field. Furthermore, apart from the latest research findings, changes in the welfare system can also impact decisions about health. The individuals and families may be influenced worst by the new principles in social security. Hence, with proper HL, the people may be able to follow and comprehend the meaning of changes from their own social situation. The current review shows that HL is a complex and depends upon the context. Therefore, there is a need to be creative in designing interventions and methods to increase and stabilize HL levels of people. Health literate people will have a better understanding of health system, greater opportunities and abilities to make decisions which may positively affect their health.

Future research needs to overcome these deficiencies by addressing health-related knowledge of consumers, focusing on language and cultural barriers in accessing health related information, formulating strategies to incorporate HL screening in practice and increasing utilization of health-related information amongst health care providers, before imparting knowledge to consumers. This review has brought forth a lack of consistency and comparability in empirical studies exploring instruments of HL and health numeracy with sound psychometric properties as well as highlights the absence of HL in consumers specifically in Australia. This deficiency is problematic as it prevents appraisal of findings of research studies and challenges their external validity. The relationship between low levels of HL and absence of social empowerment and efficacy has been confirmed [120]. This scoping review suggests that research studies investigating HL in Australia should focus on designing new measures to assess the HL of the consumer as well as HL skills of the health care provider, health care system, and how the health care messages are disseminated to the public. To date, the scoping review reveals that scientific bodies have paid little attention to developing standardized measures to assess the health literacy, as well as HL skills of providers.

### Strengths and Limitations 

Using Arksey and O’Malley’s approach provided a robust framework for this scoping review of HL research studies carried out in Australia. Using these rigorous procedures produced a wide range of HL research. However, the purpose of a scoping review does not include to systematically combining the results of earlier studies or appraising the quality of the evidence. To minimize errors in reporting the results of the scoping review, the data entered in all tables were checked twice by the first author of the review in order to confirm the correctness and to ensure correctness and comprehensiveness, all the authors reviewed the tables and necessary modifications were incorporated.

We only included those research studies which directly mentioned the term HL and those conducted in Australia. Furthermore, some studies were conducted over 10 years ago which may have influenced the measurement tools used. Nevertheless, to ensure the specificity and selectivity of the included studies, at least two authors were involved in analyzing the content of the included studies.

## 5. Conclusions

While many have been used, there is a dearth of measurement tools with sound psychometric properties to measure HL. Globally, the low level of HL in the population is a grave problem requiring innovative and proactive initiatives. The findings from this scoping review highlight that consumers’ levels of HL are still very low in Australia, which is influencing their health behaviours, service utilization and proving burdensome on health-care system. More recent developments have tried to integrate other aspects deemed vital, including introduction of applications to increase HL and exploring HL in Aboriginal communities. The integration of these aspects should increase the accumulated knowledge related to HL, however, extensive work is needed to overcome cultural and language barriers in sharing health-related information. In addition, further work is required to explore health numeracy and develop specific populations’ tools to guarantee suitability and cultural competence. Furthermore, it is recommended that the researchers focus on overcoming knowledge deficiencies, find out risk and/or be sensitive enough to measure changes occurring due to educational strategies. Some studies have shown the influence of interventions to augment HL in Australia. Therefore, the health professionals and researchers may focus on designing a HL study using innovative and new methodologies rather than relying on the traditional methods used in the past studies. For this purpose, there is a need for the scientific community to become aware of the issues related to making information and services accessible and interacting optimally with consumers.

The agenda for increasing health information would abet the efforts of government, services and consumer and organisations to fulfill the literacy requirements in a more thorough and systematic way. This will enhance the quality and eventually the participation by consumers of healthcare system and caregivers in health decision making and self-management.

The findings of this review paper can be utilized by consumers of healthcare system, healthcare service providers, administrators, board members and everyone else to assist in enhancing their awareness about HL and inform decision-making about what they, and their organisations, could do to improve HL needs. Findings can also be used by policy makers and managers.

HL should be considered as part of a systems-level approach. This will comprise designing and launching systems and policies at an organizational level, as well as societal level to ensure that the systems respond to health literacy needs. Some examples of such systems include: varying funding mechanisms to promote action on HL; launching strategies which give priority to HL in program planning; and developing healthcare organisations in such a way that removes complexity and promotes navigation and ease of access.

Effective communication (e.g., print, electronic or other communication that is suitable for the needs of consumers) may also assist in meeting HL needs. This includes partnerships, transfer of information and interpersonal relationships between consumers and providers of healthcare system, managers, executive staff and others. The incorporation of HL into education by initiating health enhancing and education strategies, school HL campaigns and social marketing activities, along with education and training of healthcare providers, will also assist to create supportive environments.

A national approach to HL through coordinated and collaborative action in Australia has the potential to enhance the safety and quality of health care. Efforts at national, state and territory, regional and local levels (e.g., health, social, welfare and education sectors) may ensure enhanced and improved HL.

HL specifically in Australia, and generally has a potential for further research as many aspects of HL are unexplored. Future research may include indigenous culture in studies of HL and design continued assessments for capturing the broadness of skills, agents and key outcomes of HL.

## Figures and Tables

**Table 1:** Databases searched for the scoping review.

Database Type	Database	Temporal Period Covered (Start Date Reflects the Year in Which Each Database was Established. End Date is the Date in Which the Search was Limited to)
Academic	MEDLINE	1946–8 August 2018
Academic	Embase	1980–8 August 2018
Academic	CINAHL	1982–8 August 2018
Academic	Eric	1966–8 August 2018

**Table 2:** Inclusion and exclusion criteria.

Criterion	Inclusion	Exclusion
Time period	1946 to August 2018	Any study outside these dates
Language	English	Non-English
Type of article	Original research article or editorials published in a peer reviewed journal.	Any publication that was not original research, systematic reviews/meta-analysis, unpublished research. For example, PhD theses and reports were excluded.
Study focus	Articles that directly mentioned the term health literacy.	Nil
Geographical area of interest	Only those studies which were carried out in Australia.	Studies other than those which were carried out in Australia.
Setting	Any	Nil

**Table 3:** Characteristics of the selected studies.

Sr. No	Authors, Year	Objective	Study Population	Sample Size	Assessment Measure	Study Design and Analyses	Summary of Findings	Study’s Definition of HL, Constructs or Domains/Theoretical Framework
1.	(Porteous, Palmer, & Wilkinson, 2014) [76]	To gather knowledge on eating behaviours, and nutrition-related needs during pregnancy and postnatally of women admitted to the postnatal ward.	Women aged at least 18 years who were admitted to the postnatal maternity ward at a hospital	*N* = 309 women	--------------------	**Study design:** Cross-sectional study **Analysis:** SPSS, Descriptive statistics, Chi-square and independent samples *t*-tests	Many women reported an interest in nutrition and healthy weight management during pregnancy and in the postnatal period and rated its importance highly, and had poor diet quality, despite identifying healthy eating as a personal priority. 42% of the women self-reported gaining excess weight during pregnancy. One quarter reported knowing their gestational weight gain goals, yet only 1.6% was correct. Half reported interest in receiving nutrition education during pregnancy and post-delivery. Nutrition topics requested included healthy eating for development of baby pre- and post-delivery and maternal weight management.	----------------
2.	(Adams, Appleton, Hill, Ruffin, & Wilson, 2009) [77]	To measure the level of functional health literacy in adults with asthma from a representative population sample and explore associations with health status and morbidity.	In a non-replacement sample, 1 adult age 15 years or older, having birthday in near future, was selected for interview in the home by trained health interviewers.	*N* = 2824 Males = 1358 Females = 1466	Newest Vital Sign (a screening test developed specifically for use in primary care)	**Study design:** Data were obtained from the South Australian Health Omnibus Survey during spring 2008. **Analysis:** SPSS, Bivariate associations with Chi-square tests, Multiple logistic regression models	Inadequate and at-risk functional health literacy is common and is associated with adverse asthma outcomes in a representative sample of adults with asthma. Increased symptoms, functional impairment, and significant health care use were more common in people with less health literacy in analyses adjusted for age, sex, and education	--------------------
3.	(Maguire et al., 2011) [78]	The aim of this study was to explore health information sources accessed by people with schizophrenia and the level of trust invested in them.	People with schizophrenia, general practice attendees	*N* = 309 71 = adults with schizophrenia (recruited from both community and inpatient settings) 238 = general practice attendees.	----------------	Study design: Cross-sectional survey, questionnaire, Analysis: SPSS version 17, independent *t*-test, Chi-square test, Mann-Whitney *U* test and binary logistic regression, Correlation	There are significant differences in the reported utilization and trust of health information sources between people with schizophrenia and attendees at general practice settings. Those with schizophrenia are less likely to trust and obtain information from a doctor, and less likely to access the Internet	----------------
4.	(Oh, Jorm, & Wright, 2009) [79]	To compare young people’s preference for a website with self-help books and two face-to-face services—counselling and mental health services. To explore factors associated with believing in the perceived helpfulness of each intervention.	Young Australians aged 12–25 years	*N* = 3746	----------------	**Study design:** Telephone survey **Analysis:** SPSS version 16.0; percentage frequencies, logistic regressions	Most young people are open to the idea of accessing mental health information online, especially for disorders that are often perceived as behavioural problems. These young people also believe in helpseeking in general and are more willing to associate with peers who have mental health problems.	----------------
5.	(Eckert, Kutek, Dunn, Air, & Goldney, 2010) [80]	To examine rural and urban differences in depression-related mental health literacy, experience of depression and help-seeking.	South Australian rural and urban young men aged between 15 and 30 years, who participated in two South Australian (SA) cross-sectional population-based Health Omnibus surveys (SAHOS), conducted in the autumns of 1998 and 2008	3010 = year 1998 3034 = year 2008	Questionnaire devised by Jorm et al. which includes a vignette depicting a man (John) with classical features of depression.	Study design: Cross-sectional population-based survey Analysis: SPSS, StataSE, Pearson’s Chi-square tests, Fisher Exac*t* test	Recognition of depression increased significantly in rural and urban young men between 1998 and 2008. More rural young men than urban men identified symptoms of depression in 1998 but that was not evident in 2008. Both groups were more likely to have a close friend experience symptoms of depression and to use antidepressant medications in 2008 compared with 1998. Rural young men experienced a significant increase in recognition of personal depressive symptoms and levels of confidence in psychiatrists and psychologists in 2008 compared with 1998. Both rural and urban young men were significantly less likely to rate dealing with problems on their own as helpful in 2008 as in 1998.	----------------
6.	(Crawford et al., 2015) [81]	To determine the efficacy of the Mental Health First Aid (MHFA) training for university nursing students	Undergraduate nursing students at a large university located in Perth, Western Australia	*N* = 126 63 = intervention group 63 = control group	----------------	**Study design:** Experimental, Randomised Controlled Trial **Analysis:** SPSS Version 22; independent samples *t*-test or Pearson’s Chi-square test, Repeated measures analysis of variance, Logistic regression	Given the known burden of mental health disorders among student populations, it is important universities consider effective strategies to address mental health issues. Providing Mental Health First Aid (MHFA) training to students offers the advantage of increasing mental health literacy, among the student population. Further, students trained in MHFA are likely to utilise these skills in the broader community, when they graduate to the workforce. It is anticipated that this trial will demonstrate the scalability of MHFA in the university environment for pre-service nurses and that implementation of MHFA courses, with comprehensive evaluation, could yield positive improvements in the mental health literacy amongst this target group as well as other tertiary student groups	Mental health literacy incorporates several components: “(a) the ability to recognise specific disorders or different types of psychological distress; (b) knowledge and beliefs about risk factors and causes; (c) knowledge and beliefs about self-help interventions; (d) knowledge and beliefs about professional help available; (e) attitudes which facilitate recognition and appropriate help-seeking; and (f) knowledge of how to seek mental health information” (Jorm, 2000) [82].
7.	(Smith, Petrak, Dhillon, Taylor, & Milross, 2014) [83]	This study aimed to (1) explore radiation oncologists’ understandings and awareness of health literacy among patients with a reasonable command of English; (2) gain insight into oncologists’ views regarding health literacy; and (3) identify techniques oncologists employ to communicate to different literacy populations.	Radiation oncologists	*N* = 26 radiation oncologists. 19 = male 7 = female	----------------	**Study design:** Qualitative, Semi-structured interviews **Analysis:** Framework method	Four key themes were identified: (1) identifying a patient’s literacy level; (2) perceived impact of literacy; (3) challenges and strategies to communicating concepts and supporting decision-making; and (4) suggested improvements to the health system. Participants described subjectively assessing a person’s literacy level by monitoring the types of questions asked; analysing the language used; examining non-verbal behaviour, and considering a person’s socio-economic situation. Participants reported the challenges of discussing the subtleties of cancer treatments with lower literacy groups such as the benefits and risks of treatment options and clinical trials, and tended to provide the basic facts to facilitate understanding.	Health literacy represents ‘the cognitive and social skills which determine the motivation and ability of individuals to gain access to, understand and use information in ways which promote and maintain good health’ (Nutbeam 2000) [84].
							Radiation oncologists acknowledged the importance of health literacy in oncology, and employed a number of techniques to tailor their communication to different literacy populations.	
8.	(Rees, Austen, Anderson, & Egan, 2014) [85]	This study investigated the impact of providing corrective information about the nature of intrusive thoughts on their subsequent appraisal in a community sample.	Community participants	*N* = 148 community participants	Obsessive Compulsive Inventory-Revised, Intrusions Inventory	**Study design:** Experimental design **Analysis:** Descriptive statistics and mean comparisons, Correlation analysis, Repeated measures ANOVA	The results of this study support the efficacy of provision of brief written information in reducing negative appraisals of intrusive thoughts in a community sample. It suggests a possible role for education about intrusive thoughts as a prevention strategy for obsessive-compulsive disorder.	Mental health literacy is a term used to describe an individual’s understanding of various aspects of mental illness. This can include knowledge about early warning signs for common psychological disorders, or understanding treatment options.
9.	(Isaacs, Maybery, & Gruis, 2013) [86]	To explore the help-seeking behaviour of Aboriginal men who are mentally unwell in a rural Victorian community.	Aboriginal people in Australia including men, carers and those involved in service delivery.	*N* = 17 Aboriginal people 15 = male	----------------	**Study design:** Qualitative design, semi-structured interviews **Analysis:** Thematic analysis	The findings suggest that there is a need for programmes that aim to improve mental health literacy and promote help seeking among Aboriginal men who are mentally unwell. Such programmes need to be developed jointly by mental health services as well as Aboriginal stakeholders, and implemented in a culturally sensitive and acceptable way.	----------------
10.	(Jamieson, Parker, & Richards, 2008) [87]	To investigate the social, cultural and environmental context of oral health among a group of Indigenous Australians in South Australia’s mid-north region.	Participants were Indigenous and had lived in South Australia’s mid-north region for most of their lives	*N* = 34 4 = focus group discussions 30 = females 4 = males	----------------	Study design: Qualitative design, Focus group discussions Analysis: Nud*st QSR N6 software, Thematic analysis	Five sub-categories were identified; ‘lifestyle changes’, ‘oral health behaviours’, ‘barriers to dental care’, ‘impact of poor oral health’ and ‘oral health literacy’. Participants felt that historical legacy impacted on the oral health of community members, through continued practices of being told what to do, where to live and what oral health services were available to them. Participants perceived they had little power over their oral health or oral health care decisions.	----------------
11.	(Hosking, Buchbinder, Pasco, Williams, & Brennan-Olsen, 2016) [88]	To add that health literacy is likely to play a major role in the ability of patients to access, understand, and apply information in order to make informed decisions about osteoporosis treatment.	Osteoporosis patients		Health Literacy Questionnaire (HLQ) (to assess individual’s health literacy needs)	Letter to the editor	The comprehension of health literacy needs of a person by applying most recent tools of health literacy in order to develop understanding regarding obstacles in medication. This strategy would ensure addressing problems of patients in person-centered way, improved adherence to medication and attaining outcomes of patients.	----------------
12.	(White et al., 2013) [89]	To assess the impact of a brief intervention (BI) designed to improve HCV vaccine CTL among people who inject drugs in Sydney, Australia.	People who inject drugs	*N* = 102 74 = male 28 = female	----------------	**Study design:** Experimental, Community-based prospective observational study **Analysis:** STATA 12.0; Means, medians, interquartile ranges, paired samples *t*-test, Multiple logistic regression	A significant increase in hepatitis C virus (HCV) vaccine clinical trial literacy (CTL) was observed, suggesting that new and relatively novel concepts can be learned and recalled in this group. These findings support the feasibility of future trials among this population.	----------------
13.	(Briggs et al., 2011) [90]	To measure broad elements of health literacy among individuals with chronic low back pain (CLBP) and without low back pain (LBP) using the Health Literacy Measurement Scale (HeLMS).	adults who reported either CLBP for 3 months or more, or no history of LBP within the last 12 months	*N* = 80 36 = Community-dwelling adults with chronic low back pain 44 = Community-dwelling adults wit with no history of low back pain	Nordic Musculoskeletal Pain Questionnaire, Nordic Musculoskeletal, Oswestry Disability Index, Avoidance Beliefs Questionnaire, Back Pain Beliefs Questionnaire, Coping Skills Questionnaire, Functional health Literacy (s-TOFHLA). Health Literacy Measurement Scale (HeLMS) (to measure an individual’s ability to seek, understand or utilise health information)	Study design: Community-based cohort study in Perth, Western Australia, from November 2008 to examine familial associations in LBP. Analyses: SPSS Statistics 17.0; independent *t*-tests Chi-square tests, Mann-Whitney tests	Although no differences were identified in HeLMS scores between the groups for seven of the health literacy domains, adults with CLBP reported greater difficulty in engaging in general positive health behaviours. This aspect of health literacy suggests that self-management support initiatives may benefit individuals with CLBP.	Health literacy—the ability to seek, understand and utilise health information
14.	(Smith et al., 2010) [91]	To determine whether a decision aid designed for adults with low education and literacy can support informed choice and involvement in decisions about screening for bowel cancer.	Adults aged between 55 and 64 with low educational attainment, eligible for bowel cancer screening.	*N* = 572 adults	----------------	Study design: Experimental, Randomized controlled trial Analysis: SPSS, paired sample *t*-test and Wilcoxon signed rank test, Mann-Whitney test, Chi-square test, Mantel- Haenszel test	Tailored decision support information can be effective in supporting informed choices and greater involvement in decisions about faecal occult blood testing among adults with low levels of education, without increasing anxiety or worry about developing bowel cancer. Using a decision aid to make an informed choice may, however, lead to lower uptake of screening.	----------------
15.	(Saunders & Peerson, 2010) [92]	To the editor	To the editor		----------------	Letter to the editor	Australian health initiatives aimed at reducing the burden of chronic and preventable disease should consider the role and challenges of health literacy beyond its application to health services use. The growing understanding of the complex behavioural, social, systemic, and ecological forces that influence health and wellbeing should help guide these efforts.	----------------
16.	(Jennings, Spurling, & Askew, 2014) [93]	To identify barriers and enablers to undertaking health checks in an urban Aboriginal Medical Service.	Clinical staff (doctors, nurses and Aboriginal and Torres Strait Islander health workers, AHWs)	*N* = 25 clinical staff (doctors, nurses and Aboriginal and Torres Strait Islander health workers). 10 = AHWs 8 = nurses 7 = doctors	----------------	**Study design:** Qualitative research design; Semi-structured Interviews **Analysis:** NVivo 9 software, content analysis	Data analysis revealed that successful completion of HCs was contingent upon several interconnected components, including the client attending the Aboriginal Medical Service (Shamsuddin et al.) and consenting to the Health Check (HC), and staff initiating and completing it. Barriers and potential enablers were indentified at each of these stages, in addition to overarching systems within the clinics.	----------------
17.	(Gratwick-Sarll & Bentley, 2014) [94]	To improve eating disorders mental health literacy.	undergraduate Australian National University third year psychology students	*N* = 177 undergraduate students. 141 = female 35 = male 1 = other	First Aid Experiences Questionnaire, First Aid Knowledge Test, Social Distance Scale, Mental Health Literacy Questionnaire for Bulimic Type Eating Disorders (to assess health literacy related with eating disorders)	Study design: Repeated measures, uncontrolled, preliminary evaluation of a single 3-hour workshop Analysis: Paired samples two-tailed *t*-tests, repeated measures analyses of variance (ANOVAs), McNemar’s test, Cochran’s Q	Following participation in the workshop, significant increases in eating disorder recognition and knowledge, and significant decreases in stigmatizing attitudes, were reported by participants. Moreover, 85% of participants reported that they provided assistance to someone whom they suspected had a mental health condition, including an eating disorder, during the 3-month follow-up period.	Mental health literacy: the knowledge and beliefs that individuals hold, which lead to improved recognition, management, and prevention of their own or another’s mental disorder (Jorm, Korten, Jacomb, Christensen, Rodgers, & Pollitt, 1997)
18.	(Caposecco, Hickson, Meyer, & Khan, 2016) [95]	This study investigated if a hearing aid user guide modified using best practice principles for health literacy resulted in superior ability to perform hearing aid management tasks, compared with the user guide in the original form.	Participants having ages 55 years or older, living in the community, comfortable speaking and reading English, and have no experience using or managing hearing aids (HAs).	*N* = 89 adults ages 55 years and over. 47 = Modified guide 42 = The original guide	Demographic Questionnaire, The Measure of Audiologic Rehabilitation Self-Efficacy for Hearing Aids, The Hearing Aid Managemen*t* test, The Shor*t* test of Functional Health Literacy in Adults (Timed reading comprehension test that consists of two prose passages containing actual materials that an adult might encounter in a healthcare setting e.g., instructions for a gastrointestinal procedure), Montreal Cognitive Assessment, Grooved Pegboard Test, Pure-Tone Audiogram	**Study design:** Experimental, Two-arm study design. **Analysis:** Stata software, independent samples *t* test, Chi-square test, Mann–Whitney *U* test, and the Fisher’s exac*t* test, multivariable linear regression model, multivariable linear regression model	Findings indicate that the need to design hearing aid user guides in line with best practice principles of health literacy as a means of facilitating improved hearing aid management in older adults.	Health literacy refers to “the degree to which individuals have the capacity to obtain, process, and understand basic health information and services needed to make appropriate health decisions” (Ratzan & Parker 2000, p. 3).
19.	(Cashin, Heartfield, Cox, Dunn, & Stasa, 2015) [96]	This paper presents analysis of consumer focus groups that were undertaken as a part of the project to develop the now current Nursing and Midwifery Board of Australia’s Nurse Practitioner Standards for Practice.	Consumer. The age of the consumers varied from people in their early 20s to people who were over 60 years of age.	*N* = 6 focus groups (32 consumers)	----------------	Study design: Qualitative, focus groups Analysis: Interpretive analysis	Consumers’ knowledge of nurses’ roles in the Australian primary healthcare system, and hence system literacy (particularly in terms of navigating the system), was low. Of perhaps greatest importance is the fact that those consumers with low health systems literacy also exhibited a low level of motivation to seek new knowledge. Many consumers relied on the medical profession to direct care.	Health literacy relates not only to individual literacy skills, but also to knowledge of the healthcare context or system Australian Commission on Safety and Quality in Healthcare (ACSQHC 2013).
20.	(Davies, Bukulatjpi, Sharma, Davis, & Johnston, 2014) [97]	To explore the knowledge, perceptions and experiences of remote dwelling Indigenous adults and their health care providers relating to hepatitis B infection with a view to using this as the evidence base to develop a culturally appropriate educational tool.	Health clinic staff, community health educators, liver clinic staff—both urban and remote, —and doctors and nurses, Indigenous and non-Indigenous, Indigenous people living with chronic hepatitis B (CHB) and Indigenous community members.	12 = patients with hepatitis B 9 = community members 13 = key informants 25 = were Indigenous individuals	----------------	**Study design:** Participatory action research project design, Semi-structured interviews **Analysis:** Deductive and inductive thematic analysis	Low levels of biomedical knowledge about Hepatitis B, negative perceptions of Hepatitis B, communication (particularly language) and culture were the major themes that emerged from the data. Accurate concepts grounded in Indigenous culture such as “only your blood can tell the story” were present but accompanied by a feeling of disempowerment due to perceived lack of “medical” understanding, and informed partnerships between caregiver and patient. Culturally appropriate discussions in a patient’s first language using visual aids were identified as vital to improving communication.	Paasche-Orlow & Wolf’s model of the pathways linking health literacy and health outcomes (Paasche-Orlow, & Wolf, 2007)
21.	(Yuen et al., 2016) [98]	To develop a conceptual model that describes the elements of cancer caregiver health literacy.	Caregivers, people with cancer and healthcare providers/policymakers.	*N* = 6 concept mapping workshops were conducted 13 = caregivers 13 = people with cancer 11 = healthcare providers/policymakers	----------------	Study design: Mixed methods approach; Concept mapping	Six major themes and 17 subthemes were identified from 279 statements generated by participants during concept mapping workshops. Major themes included: access to information, understanding of information, relationship with healthcare providers, relationship with the care recipient, managing challenges of caregiving and support systems. The study extends conceptualisations of health literacy by identifying factors specific to caregiving within the cancer context. The findings demonstrate that caregiver health literacy is multidimensional, includes a broad range of individual and interpersonal elements, and is influenced by broader healthcare system and community factors.	Health literacy can be understood as a range of ‘personal characteristics and social resources needed for individuals and communities to access, understand, appraise and use information and services to make decisions about health, or that have implications for health. Health literacy includes the capacity to communicate, assert and enact these decisions’ (Yuen et al. 2014) [98].
22.	(Nasuuna, Santoro, Kremer, & de Silva, 2016) [99]	To examine the relationship between health conditions, specialist health service utilisation and academic performance in Australian children.	Victorian children	*N* = 24,678 children 12,660 = men 11,982 = women	School Entrant Health Questionnaire (survey tool with parent report on children’s health)	**Study design:** Prospective quasi-longitudinal study **Analysis:** Univariable and multivariable linear regression, Linear and logistic regressions	Some health conditions put children at risk of poorer academic performance, and interventions to prevent this such as appropriate support services in schools should be considered.	----------------
23.	(Whittaker, Hodge, Mares, & Rodney, 2015) [100]	To identify the minimum health information system (Akoijam, Jamir, Phesao, & Senjam) competencies that could be expected in low- and middle-income countries (LMICs) that do not have advanced technology.	Health workers	*N* = 38 experts with broad-based HIS knowledge and extensive development experience 21 = male 17 = female	----------------	Delphi approach Analysis: Qualtrics^®^ software and content analysis.	Based on the initial competencies identified in the literature review and after two rounds of consultation with experts via a Delphi method, 68 HIS competencies (51 core and 17 ICT-specific) were identified in this consultation. The competencies focused on both the generation and use of data at all levels of the health system, highlighting the importance of embedding a culture of information use. This consultation is one of the first to identify the HIS competencies required among general health workers, as opposed to specialist HIS roles. It is also one of the first attempts to develop a framework on minimum HIS competencies needed in LMICs, highlighting the skills needed at each level of the system, and identifying potential gaps in current training to allow a more systematic approach to HIS capacity-building.	----------------
24.	(May, Rapee, Coello, Momartin, & Aroche, 2014) [101]	To investigate differences in mental health knowledge and beliefs between participants from the Iraqi and Sudanese refugee communities, and Australian born individuals, in Sydney, Australia.	Adults (18 years and over); Iraqi nationals, Sudanese nationals, and Australians.	*N* = 97 32 = Iraqi nationals 32 = Sudanese nationals 33 = Australians.	----------------	**Study design:** Mixed-method, semi-structured interview, Chi-square test, one-way ANOVA **Analysis:** SPSS, Thematic analysis	Although sampling was non-random, suggesting caution in the interpretation of results, it appears that the mental health literacy of lay Australians may be more aligned with the western medical model of mental disorder than that of Iraqi and Sudanese refugee communities. Mental health literacy support needs of Iraqi and Sudanese refugee communities resettled in western countries such as Australia might include education about specific symptoms and causes of mental disorder and the effectiveness of psychiatric treatments.	Mental health literacy refers to the knowledge and beliefs about mental disorders which aid their recognition, management, and prevention (Jorm, 2000) [82].
25.	(Simpson et al., 2015) [102]	To evaluate the sexual health literacy among students at the University of Tasmania.	University student populations	*N* = 1786	University of Missouri Sexual Health Survey (having three domains i.e. knowledge, sexually transmissible infection and pregnancy), ARCSHS Secondary Students and Sexual Health Survey having two domains (i.e. knowledge and HIV/Hepatitis)	Study design: Cross-sectional study Analysis: STATA/SE 12.0 for Windows (StataCorp, College Station, TX, USA); Linear regression	This study, one of the first among university students in Australia, found a varied SHL by sex, age, sexual education and sexual experience, as well as by birthplace and religious affiliation. These findings have applications in orientation and education programs at Australian universities.	Sexual health literacy (SHL) is the knowledge and familiarity with healthy practices as regards sexual health, and risk reduction strategies to engage in sexual activity safely and minimise negative consequences
26.	(Yiu, & Bajorek, 2018) [103]	To (1) characterise older patients taking warfarin, (2) assess these patients’ level of warfarin knowledge, and (3) describe their strengths and limitations in health literacy, and (4) explore relationships between participants’ characteristics, warfarin knowledge and health literacy.	older patients (aged >65 years) taking warfarin in an Australian general practice setting	*N* = 34	Purpose-designed questionnaire to record participants’ medical history, medication history, history of warfarin use, previous education received about warfarin Customised brief Warfarin Knowledge Questionnaire Health Literacy Questionnaire (HLQ	Descriptive, questionnaire-based pilot study, IBM SPSS version 23.0, Microsoft Excel. Descriptive statistics, non-parametric tests, e.g., chi-square test, Mann-Whitney *U* test, Spearman correlation test	In this study warfarin knowledge gaps and a limitation of health literacy amongst a small sample of older patients were identified. The findings suggest that education and resources may need to be tailored to the needs of older patients taking warfarin and their carers to address these knowledge gaps and limitations in health literacy. Patients who may need greater support include those that need assistance in completing the HLQ, are overseas-born, or are taking 5 or more long-term medications.	World Health Organisation (WHO) definition: the cognitive and social skills which determine the motivation and ability of individuals to gain access to, understand and use information in ways which promote and maintain good health [27]
27.	(Stanton, Rebar, & Rosenbaum, 2018) [104]	To examine the community’s mental health literacy, and views regarding exercise delivery for people with depression.	People with depression	*N* = 1265	Questionnaire devised by Jorm et al. [82] which includes a vignette depicting a man (John) with classical features of depression	telephone-based 2017 National Social Survey, cross-sectional population survey, descriptive statistics, Chi-square tests for goodness of fit	Australian adults demonstrate a high level of exercise and mental health literacy. The high level of support for accredited exercise physiologists is evidence of the effectiveness of health promotion campaigns from peak exercise professional agencies.	-------------------

**Table 4:** Spatial and temporal sequence of work done.

Sr. No.	Author, Year	Broader Region (State Wise)	Geographical Location		Time Sequence	
			Data Collected From (Location)	Corresponding Author’s Institutions	Time Frame of Data Collection	Study Published on
1.	(Porteous et al., 2014) [76]	Queensland	Socially disadvantaged area approx. 28 km south of Brisbane (Queensland, Australia)	Logan Hospital, Queensland Health, Meadowbrook, Australia.	Between 18th May 2012 and 8th October 2012	2014
2.	(Adams et al., 2009) [77]	South Australia	-------------------	University of Adelaide	South Australian Health Omnibus Survey during spring 2008	2009
3.	(Maguire et al., 2011) [78]	Australian Capital Territory (ACT)	Australian Capital Territory (ACT)	Australian National University, Canberra Hospital, Woden, Australian Capital Territory, Australia.	July to October 2009	2011
4.	(Oh et al., 2009) [79]	Whole Australia	Covering the whole Australia	University of Melbourne Locked Bag 10 Parkville (Services) 3052, Australia	June to August 2006	2009
5.	(Eckert et al., 2010) [80]	South Australia	South Australia	University of Adelaide, Adelaide, South Australia.	1998 and 2008	2010
6.	(Crawford et al., 2015) [81]	Western Australia	Perth, Western Australia	Curtin University, Perth, Australia		2015
7.	(Smith et al., 2014) [83]	New South Wales	-------------------	University of New South Wales, Sydney, Australia	July and December 2011	2014
8.	(Rees et al., 2014) [85]	Western Australia	-------------------	Curtin University, Perth, Western Australia	-------------------	2014
9.	(Isaacs et al., 2013) [86]	Victoria		Monash University, Australia.	-------------------	2013
10.	(Jamieson et al., 2008) [87]	Indigenous Australians	rural-dwelling Indigenous Australians; South Australia (those identifying as Aboriginal, Torres Strait Islander or both) Port Augusta and surrounding areas	University of Adelaide, Australia	-------------------	2007
11.	(Hosking et al., 2016) [88]	Victoria	-------------------	Deakin University, Geelong, Australia	-------------------	2016
12.	(White et al., 2013) [89]	New South Wales	Sydney, Australia.	University of New South Wales, Sydney, Australia.	November 2008 and September 2010	2013
13.	(Briggs et al., 2011) [90]	Western Australia	Perth, Western Australia	Curtin University, Australia	November 2008	2011
14.	(Smith et al., 2010) [91]	New South Wales	New South Wales, Australia	Sydney School of Public Health, University of Sydney, Australia	-------------------	2010
15.	(Saunders & Peerson, 2010) [92]	Victoria	-------------------	Deakin University, Geelong, Australia	-------------------	2010
16.	(Jennings et al., 2014) [93]	Queensland	Community-controlled Aboriginal and Torres Strait Islander medical services (AMSs) servicing Brisbane, Australia	The University of Queensland, Herston, Australia.	November and December 2010.	2014
17.	(Gratwick-Sarll & Bentley, 2014) [94]	ACT	Australian National University	Australian National University, Canberra, Australia	-------------------	2014
18.	(Caposecco et al., 2016) [95]	Queensland	-------------------	The University of Queensland, Brisbane, Australia.	-------------------	2016
19.	(Cashin et al., 2015) [96]	Western Australia, New South Wales, Queensland, Australian capital city, South Australia, Northern Territory	Southern Cross University and the University of Sydney. The focus groups occurred in: Rockingham, a small city south of Perth in Western Australia; the regional city of Cowra in New South Wales; Southport on the Gold Coast of Queensland; Darwin, the capital city of the Northern Territory; Canberra, the Australian capital city; and Adelaide, the capital city of South Australia.		2012	2015
20.	(Davies et al., 2014) [97]	Northern Territory	Northern Australia; health clinic of a remote community in Arnhem Land, 521 km northeast of Darwin (the	Menzies School of Health Research, Rocklands Drive, Tiwi, Darwin, Australia	July 2012 and December 2013.	2014
21.	(Yuen et al., 2016) [98]	Victoria	Two chemotherapy clinics from one public health service in Melbourne, Australia	Deakin University, Burwood, Australia	-------------------	2016
22.	(Nasuuna et al., 2016) [99]	Victoria	-------------------	The University of Melbourne, Parkville, Australia.	Fifty-six percent of 2008 School Entry Health Questionnaire (SEHQ) was linked to the year 3 National Assessment Program—Literacy and Numeracy (NAPLAN) collected in 2011	2016
23.	(Whittaker et al., 2015) [100]	Queensland	Science Direct, Pub Med, CINAHL, Google Scholar, Internet search engine (google.com)	The University of Queensland, Brisbane, Australia	Database searches covered the period 1990–2012.	2015
24.	(May et al., 2014) [101]	New South Wales	Sydney, Australia	Macquarie University, Sydney, Australia	---------------------	2014
25.	(Simpson et al., 2015) [102]	Tasmania	University of Tasmania	University of Tasmania, Australia	August/September 2013	2015
26.	(Yiu, & Bajorek, 2018) [103]	New South Wales	The Hills Shire (Greater Western Sydney)	Graduate School of Health, University of Technology of Sydney. Ultimo, NSW (Australia).	September 2015 and January 2016.	2018
27.	(Stanton, Rebar & Rosenbaum, 2018) [104]	Queensland	Population Research Laboratory (PRL) at Central Queensland University, Australia.	School of Health, Medical and Applied Sciences, Central Queensland University	July 17 and finished on 23 August 2017	2018

## Supplementary Materials

The following are available online at https://www.mdpi.com/1660-4601/16/7/1112/s1, PRISMA 2009 checklist.
