# Supplementary material for: Health Literacy Studies Conducted in Australia: A Scoping Review
Source: Int J Environ Res Public Health. 2019 Mar 28;16(7):1112. doi: 10.3390/ijerph16071112 (PMC6479782; doi:10.3390/ijerph16071112)
Supplement: Supplementary file 1 [file ijerph-16-01112-s001.pdf]

PRISMA 2009 Checklist

Section/topic	#	Checklist item	Reported on page #
**TITLE**			
Title	1	Health Literacy Studies Conducted in Australia: A Scoping Review	1
**ABSTRACT**			
Structured summary	2	Background: Health literacy (HL) is an essential component of various literacies mentioned in the field of health and education, including cultural, technological, media and scientific literacies. It is important for motivating higher consumer engagement. Objectives: We aimed to review previous studies of HL in Australia to inform future studies, extend current knowledge and further enhance HL. Method: Using search strings, a systematic search of four databases (i.e. MEDLINE; Embase; CINAHL and Eric) was carried out. Results: A Preferred Reporting Items for Systematic Reviews and Meta-Analyses (PRISMA) based search strategy led to identification of a total of N = 9696 records, that were further screened for inclusion in the review. Conclusion: The review revealed three major themes: 1) HL and health numeracy; 2) contrast of: knowledge deficiency, knowledge gained, problems of current health care system and 3) HL measurement methods and its domains. The findings from this scoping review show a dearth of measurement tools with sound psychometric properties for assessing HL. The findings also reveal low levels of HL in consumers which is in turn affecting health-related behaviors, utilization of health services and navigation of the health system. More recent developments have tried to integrate vital aspects, including introduction of applications to increase HL and exploring HL in Aboriginal communities	2
**INTRODUCTION**			
Rationale	3	This scoping review augments current knowledge on HL as well as aims to offer clarity in various concepts related with HL and highlights how HL has advanced with the passage of time region-wise (temporal and spatial), in Australia. There is a need to carry out a scoping review to analyze the research on HL due to various reasons. Firstly, regarding HL, variability exists in scientific community about its conceptual understanding, terminology, and there are improper definitions of HL in health and medical research. Secondly, there is difficulty in assessing concepts related with HL such as, interpersonal communication, quality, individual's motivation, empowerment, and decision making. Thirdly, low HL or lack of adequate levels of HL have been linked with various adverse outcomes, such as, greater utilization of services of health, low medication adherence, less information in consumers, equity issues for disadvantaged groups of Australia, and worsened health outcomes. Keeping in view these aforementioned reasons, this project aims at reviewing, analyzing and summarizing previous work done in Australia so that future studies can build on the current knowledge to further enhance HL. This project demonstrates that a decline in health literacy also obstructs a consumer's ability to deal with questions of numeracy related with intake of medicines, his/her ability to appropriately carry out instructions related to medications, and his/her ability to account any other medications he/she is consuming..	6,7
Objectives	4	to explore the prevailing awareness regarding HL in the published literature.<br>to understand the prevailing definitions and concepts of HL, a lack of standardization in measurement tools and agreement on what needs to be measured (e.g. key outcomes), as reported in the Australian empirical studies, for developing an understanding around assessment of HL and its domains	6
**METHODS**			
Protocol and registration	5	Not applicable	N/A
Eligibility criteria	6	**Please see Table 2.** Inclusion and exclusion criteria	10

Information sources	7	A systematic search of four databases MEDLINE; Embase; CINAHL and Eric was carried out before Aug 2018.	8
Search	8	For every database, the terms that related to HL, and treatment decision were extracted in order to capture the three unique levels (i.e. basic or functional level, communicative or interactional level, and critical level) of HL.	8
Study selection	9	Identification, screening of title and abstract, full text eligibility assessment, included in scoping review.	8,9
Data collection process	10	The literature as collected using MEDLINE; Embase; CINAHL and Eric databases.	8
Data items	11	The literature relating to health literacy in Australia till August 2018 was included in this study.<br>**Please see Table 1:** Databases searched for the scoping review	9
Risk of bias in individual studies	12	It was not conducted in this review as this review comprised cross-sectional studies, qualitative studies, mixed-methods studies, longitudinal study, and descriptive study. Additionally, meta-analysis was not carried out for this review.	N/A
Summary measures	13	Not applicable because we do not perform meta-analysis for this study	N/A
Synthesis of results	14	The empirical relationships relevant for answering the research question are reported and thematically organized. The first author analysed, organized, and synthesized the data comprehensively through thematic analysis. The analysis was then discussed with all the co-authors and the categories were identified. Approximately, half of the thematic analysis was autonomously carried out by the two authors and all differences were settled through discussion between two and all the authors. The results are presented narratively.	10-11
Section/topic	#	Checklist item	Reported on page #
Risk of bias across studies	15	Not applicable because we do not perform meta-analysis for this study	N/A
Additional analyses	16	Not applicable because we do not perform meta-analysis for this study	N/A
**RESULTS**			

Study selection	17	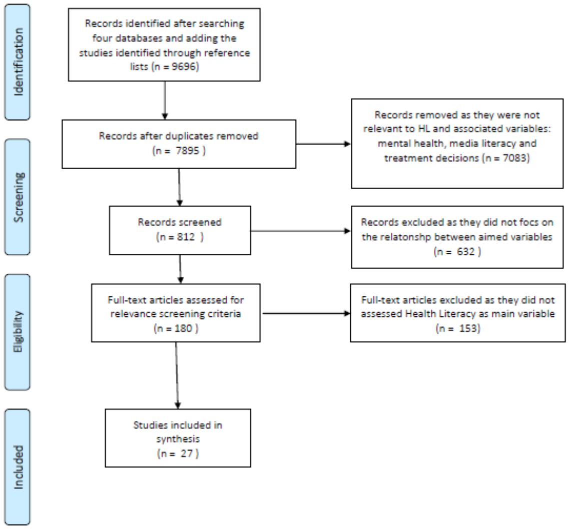	9
Study characteristics	18	**Please see Table 3.** Characteristics of the selected studies	10
Risk of bias within studies	19	Not applicable	N/A
Results of individual studies	20	Not applicable	N/A
Synthesis of results	21	We identified three main themes from the collected data: 1) HL and health numeracy 2) HL measurement methods and its domains and 3) HL as knowledge deficiency, knowledge gained knowledge of current health care system.	12-21
Risk of bias across studies	22	Not applicable	N/A
Additional analysis	23	Not applicable	N/A
**DISCUSSION**			
Summary of evidence	24	Summarize the main findings including the strength of evidence for each main outcome; consider their relevance to key groups (e.g., healthcare providers, users, and policy makers).	21-26
Limitations	25	Using Arksey and O'Malley's approach provided a robust framework for this scoping review of HL research studies carried out in Australia. Using these rigorous procedures produced a wide range of HL research. However, the purpose of a scoping review does not include to systematically combining the results of earlier studies or appraising the quality of the evidence. To minimize errors in reporting the results of the scoping review, the data entered in all tables were checked twice by the first author of the review in order to confirm the correctness and to ensure correctness and comprehensiveness, all the authors reviewed the tables and necessary modifications were incorporated.<br>We only included those research studies which directly mentioned the term HL. At least two authors were involved in analyzing the content of the included studies to ensure the specificity and selectivity of the included studies. Furthermore, some studies were conducted over 10 years ago which may have influenced the measurement tools used.	26,27

Conclusions	26	There is a dearth of measurement tools with sound psychometric properties to measure HL. Globally, the low level of HL in the population is a grave problem requiring innovative and proactive initiatives. The findings from this scoping review highlights that literature shows that the consumers' levels of HL are still very low in Australia, which is influencing their health behaviours, service utilization and proving burdensome on health-care system. More recent developments have tried to integrate other aspects deemed vital, including introduction of applications to increase HL and exploring HL in aboriginal communities. The integration of these aspects should escalate the accumulated knowledge related to HL, however, extensive work is needed to overcome cultural and language barriers in sharing health related information. In addition further work is required to explore health numeracy and develop specific populations' tools to guarantee suitability and cultural competence. Furthermore, it is recommended that the researchers may focus on overcoming knowledge deficiencies, find out risk and/or be sensitive enough to measure changes occurring due to educational strategies. Some studies have shown the influence of interventions to augment health literacy in Australia. Therefore, the health professionals and researchers may focus on designing a health literacy study using innovative and some new methodologies rather than relying on the traditional methods used in the past studies. For this purpose, there is a need that the scientific community become aware of the issues related to: standardize its practice, make information and services accessible, and interact optimally with consumers.<br>HL specifically in Australia, and generally has a potential for further research as many aspects of HL are unexplored. Future research may include indigenous culture target to investigate the health literacy and design continued assessments for capturing the broadness of skills, agents and key outcomes of health literacy.	27,28
**FUNDING**			
Funding	27	This scoping review received no funding.	39

From: Moher D, Liberati A, Tetzlaff J, Altman DG, The PRISMA Group (2009). Preferred Reporting Items for Systematic Reviews and Meta-Analyses: The PRISMA Statement. PLoS Med 6(6): e1000097. doi:10.1371/journal.pmed1000097
